# 3D organ-on-a-chip: The convergence of microphysiological systems and organoids

**DOI:** 10.3389/fcell.2022.1043117

**Published:** 2022-11-21

**Authors:** Leandra S. Baptista, Constance Porrini, Gabriela S. Kronemberger, Daniel J. Kelly, Cecile M. Perrault

**Affiliations:** ^1^ Eden Tech, Paris, France; ^2^ Universidade Federal do Rio de Janeiro, Campus UFRJ Duque de Caxias Prof Geraldo Cidade, Rio de Janeiro, Brazil; ^3^ Trinity Centre for Biomedical Engineering, Trinity Biomedical Sciences Institute, Trinity College Dublin, Dublin, Ireland; ^4^ Department of Mechanical, Manufacturing and Biomedical Engineering, School of Engineering, Trinity College Dublin, Dublin, Ireland; ^5^ Advanced Materials and Bioengineering Research Centre (AMBER), Royal College of Surgeons in Ireland and Trinity College Dublin, Dublin, Ireland; ^6^ Department of Anatomy and Regenerative Medicine, Royal College of Surgeons in Ireland, Dublin, Ireland

**Keywords:** organoids, 3D bioprinting, organ on a chip, drug development, microfluidics

## Abstract

Medicine today faces the combined challenge of an increasing number of untreatable diseases and fewer drugs reaching the clinic. While pharmaceutical companies have increased the number of drugs in early development and entering phase I of clinical trials, fewer actually successfully pass phase III and launch into the market. In fact, only 1 out of every 9 drugs entering phase I will launch. *In vitro* preclinical tests are used to predict earlier and better the potential of new drugs and thus avoid expensive clinical trial phases. The most recent developments favor 3D cell culture and human stem cell biology. These 3D humanized models known as organoids better mimic the 3D tissue architecture and physiological cell behavior of healthy and disease models, but face critical issues in production such as small-scale batches, greater costs (when compared to monolayer cultures) and reproducibility. To become the gold standard and most relevant biological model for drug discovery and development, organoid technology needs to integrate biological culture processes with advanced microtechnologies, such as microphysiological systems based on microfluidics technology. Microphysiological systems, known as organ-on-a-chip, mimic physiological conditions better than conventional cell culture models since they can emulate perfusion, mechanical and other parameters crucial for tissue and organ physiology*. In addition, they reduce labor cost and human error by supporting automated operation and reduce reagent use in miniaturized culture systems.* There is thus a clear advantage in combining organoid culture with microsystems for drug development. The main objective of this review is to address the recent advances in organoids and microphysiological systems highlighting crucial technologies for reaching a synergistic strategy, including bioprinting.

## Introduction

We are living in a world in which fewer useful drugs are discovered in a scenario of increasing untreatable diseases. Some diseases require the development of new drugs, such as antimicrobial resistance (Interagency Coordination Group on Antimicrobial Resistance. 2019), tumors and obesity, but others mainly require better, more accurate models for the prediction of toxicity (Corsini et al., 2012), a major cause of failure in drug development. The whole process of drug development is considered inefficient (e.g., 94% of drugs fail in the clinical trial phases (Paul et al., 2010; Scannell et al., 2012), leading to unsustainable costs in the healthcare system and drugs with low efficacy and safety to the population.

An analysis of hundreds of drugs that failed in the later stage of drug development found that the preliminary assays conducted in rats and dogs were only able to predict human toxicity in 71% of cases (Olson et al., 2000). The pharmaceutical industry is now questioning the quality of *in vitro* tests performed during the preclinical stage of drug development, which include 2D cell culture and animal models. Their criticisms center on the poor physiological resemblance to healthy or diseased human tissue (Zhang and Radisic, 2017), and for animal models, their lengthy time for results, high financial costs and ethical issues.

The most recent *in vitro* tests have tried to converge 3D cell culture and human stem cell biology to achieve better resemblance with the physiological system. These 3D humanized models, known as organoids, mimic the 3D tissue architecture and physiological cell behavior of healthy and diseased organs. Organoids can better predict efficacy and safety, improving the quality of preclinical tests before human clinical trials (Clevers, 2016). Furthermore, when formed from patient derived cells, they hold the potential to add valuable information to the field of personalized medicine. However, this powerful technology faces crucial issues regarding their limited small-scale production, lack of automation, costs and reproducibility, jeopardizing their translation to the pharmaceutical industry (Garreta et al., 2021). More importantly, organoids are usually cultivated in a static environment, reducing their capacity to reach differentiation.

An interesting concept has emerged to address technological limitations of organoids: the integration of basic biological knowledge of organoids with advanced microtechnologies, such as microphysiological systems based on microfluidics technology. These microphysiological systems known as organ-on-a-chip mimic physiological conditions better than conventional cell culture models since they can emulate perfusion, mechanical and other parameters crucial for tissue and organ physiology (Jang et al., 2019). However, organoid and organ-on-a-chip technologies have emerged as 3D cell culture models disconnectedly. A synergistic strategy can address limitations and add advantages coming from both technologies. For example we can combine the human cellular and tissue fidelity found in organoids and the environmental control of microfluidics chips leading to a better, more accurate technology for drug discovery and development in the pharmaceutical industry (Takebe et al., 2017). In this context, the pharmaceutical industry estimated a significant reduction in drug development costs by adopting microphysiological system technologies which could also replace animal models (Ingber, 2022). The aim of this mini-review is to address the recent advances in organoids highlighting crucial technologies for reaching a synergistic strategy with the microphysiological systems.

## Drug development: Current scenario

Drug development typically comprises four main stages: 1) discovery and development of promising compounds; 2) preclinical research using *in vitro* and *in vivo* tests; 3) clinical research and 4) application for approval by regulatory agencies. To reach approval, the novel drug must show safety and effectiveness in humans (Scannell et al., 2012). Due to the high failure rate faced today by pharmaceutical companies, the process has been revised as a whole and raised important issues of the preclinical stage (Marx et al., 2020), more specifically that preclinical results come from tests with cells of non-human origin (cell culture and animal models) and their misleading results are not replicated in clinical trials (Van Normal, 2019). The most common problems are absence of efficacy and unforeseen side effects, leading to withdrawal of drugs from the market (Jang et al., 2019).

Governments and public administrations are now under increasing pressure to find alternatives to animal testing. Already, the US Senate approved the Humane Research and Testing Act (HR 1744) and the US Food and Drug Administration (FDA) Modernization Act of 2021, a bill to amend the Federal Food, Drug, and Cosmetic Act that will allow drug manufacturers and sponsors to apply for market approval with safety and effectiveness results from alternative methods to animal testing. In the current draft of the bill, the alternative methods cited specifically include “cell-based assays, organ chips and microphysiological systems, computer modeling, and other human biology-based test methods” (Congress.Gov. 2021).

Simultaneously, in September 2021 the European Parliament adopted a resolution that goes in the same direction. It plans actions to accelerate this transition without the use of animals in research, regulatory testing and education. This resolution invites the EU Commission, stakeholders and Member States, to develop an action plan, to reduce, refine and replace procedures with live animals. This resolution calls for a scientific discussion to foster animal welfare and to promote technological innovation (European Parliament. 2021).

## 3D Cell culture

2D cell culture and animal models have allowed us to accumulate knowledge in cellular and molecular biology, but questions inherent to human cell physiology remain unanswered. 3D cell culture models, more specifically, organoids, can recreate human 3D tissue architecture and spatially and temporally recapitulate morphogenetic events due to human stem cell differentiation (Kim et al., 2020) while animal models are not predictable models for several human diseases and physiological responses, since they are constituted by animal cells.

The consensus in the scientific literature is that a complex 3D cell culture model generated from human cells holds the potential for improving the prediction of drug development (Marx et al., 2020). In fact, a complex 3D cell culture model fills the gap between 2D cell culture and animal models, bringing cell model closer in complexity to human tissues and organs. These complex 3D models only became possible after the recent discovery of human adult stem cells (including mesenchymal cells) and human induced pluripotent stem cells (iPS), since these cells can recapitulate morphogenetic events of tissue and organ development. This intrinsic differentiation capacity is optimized in 3D cell culture models using non-adherent surfaces or matrigel, where cell-cell and cell-extracellular matrix interactions prevail. These models are currently known as organoids (Panoutsopoulos, 2021).

In addition to the requirement that organoids be derived from stem cells or primary sources, they should have at least one physiologic function from the organ of origin (Rossi et al., 2018). Even now, several organoids models have been developed, including pathological models for personalized medicine. Using iPS derived cells from patients or even primary cells from intestine or tumor biopsies, it is possible to recapitulate the disease development and genetic signatures from the disease of origin (Drost and Clevers, 2018).

Organoids can also be generated from patient-derived cells, including tumors, being truly representative of a disease state in comparison with cell lines. Using patient-derived organoids allows the discovery of new biomarkers for diseases and test drugs *in vitro* before patient treatment (personalized medicine) (Yao et al., 2020). However, complex models frequently lose their reproducibility, mandatory for the pharmaceutical industry. Furthermore, unlike 2D models, organoids are not currently compatible with high-throughput screening. For organoids to become a predictable, reproducible model suitable for use by the pharmaceutical industry, a number of challenges must be overcome: 1) high control over nutrients supply together with the biochemical and biophysical microenvironments; 2) Reduction of variability, achievable through higher use of automated protocols and 3) Better simulation of body physiology by modeling tissue-tissue and multiorgan interactions.

A promising pathway to solve these technological challenges is the integration of organoids with microphysiological systems based on microfluidics technology. Organ-on-a-chip (or organ chip) are microfluidic cell culture devices that represent one of the recent successes in the search for *in vitro* human microphysiological systems that can recapitulate organ-level and even organism-level functions. Methods based on microfluidics are used today in the development of a drug candidate. The health authorities (FDA and EMA) are in the process of carrying out a review comparing organ-on-chip technologies with conventional methods to validate their level of reliability. Recently, FDA approved a clinical trial without animal data for a rare neuromuscular disorder based on results from an organ-on-a-chip model (ZME SCIENCE. 2022).

Unlike 2D cell culture models, organ-on-a-chip mimics several physiologic parameters crucial for tissue and organ physiology (Jang et al., 2019)*.* These systems can also be connected to each other reaching a multiorgan-on-a-chip concept, where multiple organ models are interconnected by the laminar flow (Picollet-D'hahan et al., 2021). More importantly, they reduce labour cost and human error by supporting automated operation and reduce reagent use in miniaturized culture systems. A novel concept emerges—the organoid-on-a-chip and will be discussed in this mini-review.

## Organ-on-a-chip

Organ-on-a-chip technologies are based on microfluidic devices seeded with cells maintained under constant fluid flow (Ingber, 2022). The main goal of the earliest organ-on-a-chip models was to mimic crucial physiological parameters, mainly based on mechanical stimuli. The first published organ-on-a-chip model was built to simulate the alveolar-capillary interface of the human lung using epithelial and endothelial cells. The device was capable of simulating breathing-type movements and responding to pathogen stimulus (Huh et al., 2010).

Currently, those microphysiological systems come in different sizes and shapes. They contain small and hollow channels seeded with living cells. Those micro-channels have sizes comparable to blood capillaries, which provide the necessary nutrients and oxygen. That way, living tissues are cultured under dynamic flow. Microfluidic devices recreate organ structures (co-culture, interphases, 3D-organization) and mechanical forces (e.g., shear or stretch forces), conditions necessary to mimic organ physiology and processes. The biomechanical forces induced by the flow in the microchannels mimic the pressures exerted by vascularization, required for cell differentiation (Stone et al., 2004). Furthermore, it is possible to connect two or more organ-on- chips. A “body-on-chip” or “multi organ-on-a-chip” is a multi-organ system that models whole-body physiology or pathology (Picollet-D'hahan et al., 2021).

Over the last years, the main methods used to develop microfluidics devices were conventional manufacturing techniques, such as photo-patterning, self-assembly and soft lithography (Carvalho et al., 2021). However, when compared to those manufacturing techniques, 3D printing includes the advantages of unlimited design space, freedom of complex geometries and reduction of waste.

Bazaz and collaborators Bazaz et al. (2020) proposed a new method for the fabrication of inertial microfluidic devices using 3D printing. The authors achieved relevant geometries for cell behavior, such as straight, spiral, serpentine, curvilinear and contraction-expansion arrays. Another study printed the microfluidic device by extrusion fused deposition modeling technique (FDM) of transparent polymers and observed that the cells successfully adhere on the surface of the devices together with a high viability after the initial 3 days in culture (Mehta et al., 2021).

Although all advances in organ-on-chip technologies, their cellular composition and tissue architecture is simple, resembling co-culture techniques (Takebe et al., 2017). Due to the absence of stem cell population and tissue microenvironments, under a pathogen or drug stimulus, the organ-on-a-chip will not go through a tissue remodeling, which in turns limits their physiological relevance. This issue applies to all disease models, but is particularly sensitive in tumorigenesis models, as tumor biology is quite dynamic, containing various cell subpopulations at different degrees of mutation.

## Organoids recapitulating models of diseases

As 3D cell culture technologies advance, as well as our understanding of protocols for inducing morphogenesis, the complexity of organoids increases, including in the diversity of cell types used and, consequently, their function, compared to the organ of origin (Mccracken et al., 2014; Zhang et al., 2016; Achberger et al., 2019; Silva et al., 2021; Sun et al., 2021). These hallmarks are summarized in Table 1.

**TABLE 1:** Recent advances: hallmarks of organoids models.

Hallmarks	Doi	Year
Cerebral organoids recapitulate the development and disease condition of the native tissue	10.1038/nature12517	2013
Human gastric organoids representing the first complex gastric tissue *in vitro* model suitable for drug discovery	10.1038/nature13863	2014
Successful production of human lung organoids similar to human fetal lung based on transcriptional characterization	10.7554/eLife.05098	2015
Successful protocol for differentiating hPSCs into multipotent nephron progenitor cells that can form nephron-like structures containing epithelial nephron-like structures.	10.1038/nbt.3392	2015
Different stages of organoid formations require different microenvironments	10.1038/nature20168	2016
The method to produce endothelialized organoids can be applied for drug screening tests	10.1016/j.biomaterials. 2016.09.003	2016
Successful production of colorectal organoids	10.4103/0366-6999.191782	2016
Description of human whole-brain organoids with high level of maturation	10.1038/nature22047	2017
Patient-derived bladder organoids as a functional model system for studying tumor evolution and treatment	10.1016/j.cell. 2018.03.017	2018
Development of a protocol for medium-high throughput drug screening of human organoids	10.1007/7651_2016_10	2019
Successful production of retinal organoids as a model for drug development	10.7554/eLife.46188	2019
Successful establishment of a model system to engineer patient-specific glioblastoma	10.1016/j.celrep. 2019.02.063	2019
Development of human liver organoids to recapitulate steatohepatitis-like pathology	10.1016/j.cmet. 2019.05.007	2019
Successful differentiation of human iPSCs to kidney organoids	10.1038/s41592-018-0253-2	2019
Development of functional 3D brain spheroids and organoids	10.1038/s41586-019-1289-x	2019
Hepatic organoids as a model to investigate alcoholic liver disease	10.1038/s41422-019-0242-8	2019
Establishment of organoid lines from patent-derived ovarian cancer	10.1016/j.stemcr. 2020.03.004	2020
Successful development of a hepatic organoid platform with human cells to explore complex liver diseases	10.1053/j.gastro. 2020.06.010	2020
Non-small cell lung cancer organoids recapitulates the genomics and biology of patient tumors	10.1158/1078-0432.CCR-19-1376	2020
Development of a standardized approach for production of midbrain organoids	10.1016/j.scr. 2020.101870	2020
Functional brain organoids displayed early and late expression of neuronal markers	10.3390/ijms21030694	2020
Pancreatic ductal adenocarcinoma tumor organoids can be used as a platform for discovering biomarkers	10.1172/jci.insight.135544	2020
SRT1720 can be used as a new effective treatment for bladder cancer	10.1038/s41388-021-01999-9	2021
Fabrication of a new *in vitro* organoid model to study epithelial regeneration	10.1038/s41422-020-00453-x	2021
Development of a new platform for the transplantation of epithelial cells	10.1038/s41586-021-03247-2	2021
Successful development of multi-lineage/multi-organ organoids	10.1016/j.stem. 2021.11.007	2021
Papillary thyroid cancer organoids can be used as preclinical model for drug screening tests	10.1210/clinem/dgab020	2021
Synovial mesenchymal stromal cell organoids provide a model for Osteoarthritis and drug testing	10.1038/s41392-021-00675-4	2021
Successful production of kidney organoids as a tool for embryonic kidney developmental model	10.1371/journal.pone.0252156	2021
Neuroendocrine carcinoma organoids as *in vitro* models	10.3389/fendo. 2021.627819	2021
Kidney organoids as a promising model to define the complexities of vascular-nephson interactions	10.1016/j.ydbio. 2021.04.009	2021

Currently, it is possible to generate liver organoids composed not only by their parenchymal cells, the hepatocytes, but also by stromal cells such as stellate and Kupffer-like cells. This increase of cell diversity allowed the organoids recapitulate the main steps of steatohepatitis, including steatosis, inflammation, and fibrosis phenotypes (Ouchi et al., 2019; Wang et al., 2019). Furthermore, they reach a complex tissue architecture; for example a functional bile canaliculi system, capable of responding to drug stimulus (Ramli et al., 2020).

The intestinal organoid was the first organoid protocol described in scientific literature. Using adult stem cells (lgr5 positive cells) from the human intestine biopsy seeded on matrigel, these stem cells are capable of anchoring on extracellular matrix components of matrigel mimicking the epithelial polarity of intestine without a mesenchymal niche (Sato et al., 2009). Since then, the complexity of intestinal organoid has been increased for simulating hyperplastic intestinal organoids under injury (Qu et al., 2021) and for mimicking different parts of intestine (Xie and Wu, 2016) such as the small intestine colon as a regenerative strategy for short bowel syndrome (Sugimoto et al., 2021). However, the use of matrigel is a crucial impairment for its translation, since matrigel is a poorly defined animal-derived matrix causing animal protein contamination and reproducibility (lot variation) issues. Researchers are now working on development of alternative biomaterial, mainly to replace matrigel with synthetic polymers (Gjorevski et al., 2016; Garreta et al., 2021) or even with biomaterials derived from decellularized extracellular matrix (Cho et al., 2021; Kim et al., 2022). The synthetic polymers have the advantage of tunability, while the decellularized extracellular matrix shows similarity with molecular cues of native tissues.

The cerebral organoid was the first organoid described in scientific literature derived from iPS cells. This organoid recapitulates human cortical development in healthy and disease states (Lancaster et al., 2013). The subsequent articles showed a diversity of cell types related to the human cerebral cortex (Quadrato et al., 2017; Velasco et al., 2019) with a correlation of genetic variability according to donor cells (Velasco et al., 2019). Furthermore, functional aspects have been shown such as neuronal activity under light stimulation (Quadrato et al., 2017). Interestingly, the cerebral organoid was recently used as a tumor model to study invasion behavior of patient-derived stem cells from glioblastoma. The authors observed forming tumors close to patient glioblastomas (Linkous et al., 2019). Midbrain organoids are capable of recapitulating dopaminergic neuron and astrocyte differentiation, serving as a model for Parkinson disease (Chlebanowska et al., 2020; Nickels et al., 2020). The first described protocol for cerebral organoid in 2013 was time and cost consuming and since then some degree of optimization to reduce batch-batch variability has been undertaken (Nickels et al., 2020), including the use of bioengineering approaches (Lancaster et al., 2017; Nikolaev et al., 2020; Garreta et al., 2021).

Mainly due to the pandemic of COVID-19, the interest in lung organoid development considerably increased in the last 2 years (van der Vaart et al., 2021). Pioneer research in the field of lung organoids is derived from recent studies describing 3D co-culture techniques of stem cell subpopulations with endothelial cells (Lee et al., 2014). The lung organoid derived from iPS was described soon after, showing epithelial and mesenchymal compartments, together with tissue architecture similar to native lung (Dye et al., 2015; Chen et al., 2017). Interestingly, recent advances using extracellular matrix-free and chemically defined organoid culture derived from single adult human alveolar epithelial type II (AT2) cells established a reproducible lung organoid model of human distal lung infections, including COVID-19 (Salahudeen et al., 2020).

Relevant porgress was reached for kidney organoids (Morizane et al., 2015; Phipson et al., 2018; Facioli et al., 2021; Ryan et al., 2021) for tumor organoids as a model of tumorigenesis (Francies et al., 2016; Lee et al., 2018; Huang et al., 2020; Maenhoudt et al., 2020; Chen et al., 2021; Dijkstra et al., 2021; Tan et al., 2021).

## Organoids as platforms for drug development

Organoids represent a powerful tool to model human tissues and organs at the cellular and molecular levels, with applications in drug discovery, development and testing (Yoshida et al., 2020). More importantly, such predictable models can capture specific characteristics of a person’s disease, individual responses to drugs, including side-effects (Mastrangeli et al., 2019). Organoids are now considered as avatars in personalized medicine (Seidlitz et al., 2018; Frappart et al., 2020; Jacob et al., 2020; Jian et al., 2020; Saltsman et al., 2020; Shi et al., 2020; Witte et al., 2020; Bi et al., 2021; Kazama et al., 2021; Grossman et al., 2022) with the potential to contribute to reducing the high level of drug discovery failure. Recent scientific articles related to organoids and drug testing are summarized in Table 2.

**TABLE 2:** Recent advances of organoids models for drug testing.

Advantages	Doi	Year
A co-culture of human hepatocytes and hepatic stellate cells as 3D spheroids recapitulated a hepatocyte-mediated and drug-induced liver fibrosis.	10.1016/j.biomaterials. 2015.11.026	2016
Generation of colonic organoids representing populations of cells from colon rather than small intestine. The colonic organoids derived from patient-derived iPSCs replicated the mutations. The authors found that geneticin efficiently targeted the mutation and restored normal proliferation of cells.	10.1038/nm.4355	2017
Lung cancer organoids and normal bronchial organoids derived from patient tissues represented individual patients, responding to drugs based on their genomic alterations.	10.1038/s41467-019-11867-6	2019
A biobank of patient-derived pancreatic cancer organoids were exposed to extensive drug screens, revealing unique drug sensitivity profiles.	10.1073/pnas.1911273116	2019
Human gastric cancer organoid cultures showing genetic profile in accordance to their tumor of origin as well as the response to drug treatments.	10.1136/gutjnl-2017-314549	2019
Ovarian cancer organoids were expanded in culture showing correlation with morphology, genetic profile and response to drug treatment according to their tumor of origin.	10.1038/s41598-020-69488-9	2020
Patient-derived tumor organoids from different breast cancer subtypes were established showing restoration of chemosensitivity in drug resistance.	10.3390/cancers12123869	2020
Ovarian cancer organoids maintain the genomic features of the original tumor lesion and recapitulate patient response to drug treatment. Drug screening resulted in responsiveness to at least one drug for 88% of organoids.	10.1016/j.celrep. 2020.107762	2020
Non–small cell lung cancer organoids were established from primary lung patients and PDX tumor tissue. Organoids retained morphological and genetic characteristics and also the sensitivity to targeted drugs.	10.1158/1078-0432.CCR-19-1376	2020
A biobank of glioblastoma organoids was generated to test personalized therapies by correlating mutational profiles to drug response.	10.1016/j.cell. 2019.11.036	2020
Pharmacokinetic functions (membrane permeability and metabolic stability) of organoids from human intestinal epithelial cells were evaluated after their dissociation.	10.1038/s41598-020-63151-z	2020
A comparison between patient-derived xenograft tumor (PDX) and PDX-derived organoids was carried out. Organoids recapitulated morphology, protein profile and genomic alterations. A small-scale pharmacotyping platform was also established.	10.1177/2050640620905183	2020
Organoids derived from human colorectal cancer primary tumors were transplanted in the murine spleen as an *in vivo* xenograft model of liver metastases, showing preservation of protein profile.	10.1186/s12967-020-02407-8	2020
Liver organoids were generated by co-culturing primary hepatocytes with mesenchymal stem cells. Long-term survival of the primary hepatocyte organoids, as well as stable functionality, was demonstrated.	10.4252/wjsc.v12.i10.1184	2020
Hepatoblastoma tumor organoids were generated from aggressive hepatoblastoma primary tumors. Organoids recapitulate the key elements of patient tumors, including the hepatoblastoma pathophysiology, besides responding to drug stimulus.	10.3390/cancers12092668	2020
Renal tubular organoids were generated from human urine-derived stem cells showing responsiveness to acetone and cisplatin.	10.1021/acsbiomaterials.0c01468	2020
Renal cell carcinoma organoids recapitulated morphology of primary tumors as well as genetic profile exhibiting differential responses to drug treatment.	10.3892/or. 2021.8177	2021
iPS-derived alveolar organoids were generated as a model of pulmonary fibrosis. The use of this model allowed assessing anti-fibrotic mechanisms of potential drugs.	10.1038/s41420-021-00439-7	2021
iPS-derived cardiac organoids were obtained by micropatterning, showing contracting cardiomyocytes in the center surrounded by stromal cells distributed along the surface. The pattern sizes affect contraction functions. Organoids were responsive to nine pharmaceutical compounds tested to reveal embryotoxic potential of this model.	10.1016/j.stemcr. 2021.03.013	2021
Prostate cancer organoids were established using a scalable pipeline for automated seeding, drug treatment and analysis.	10.1177/24725552211020668	2021
Cerebral organoids were used for the first time as a model of screening potential drugs for human prion diseases.	10.1038/s41598-021-84689-6	2021
Human hepatocyte-like cells derived from organoids were bioprinted into porous constructs. Cell viability was maintained for up to 10 days. The bioprinted construct was responsive to an established hepatotoxic compound.	10.1002/mabi.202100327	2021
Patient-derived organoid models of endometrial and ovarian cancer tissues were established. The model recapitulated the sensitivity to relevant chemotherapeutic agents and predicted postoperative chemotherapy of one patient.	10.3390/cancers13122901	2021
Organoids derived from pancreatic ductal adenocarcinoma showed correlation of drug response with clinical treatment in individual patients.	10.1158/1078-0432.CCR-20-4116	2022
iPS-derived hepatic organoids were established showing a high drug metabolic activity. Organoids showed remarkable CYP450 activity and recapitulated the metabolic clearance, CYP450-mediated drug toxicity, and metabolism.	10.1016/j.biomaterials. 2022.121575	2022
iPS-derived lung organoid was infected with SARS-CoV-2 as a model of drug testing. The potential drugs caused a significant reduction of viral entry and a modulation of genes involved in innate immunity and inflammatory response.	10.3390/cells11071235	2022

Tumor organoids or tumoroids have been extensively used for tumor cell expansion in culture. These tumoroids maintain cellular and genetic heterogeneity from their tumor of origin and are considered as avatars for precision cancer medicine. Several studies have demonstrated maintenance of the genomic alterations from the original tumor during long-term culture (Choo et al., 2021), including those from ovarian cancer (Nanki et al., 2020), lung cancer (Kim et al., 2019), breast cancer (Campaner et al., 2020) and pancreatic cancer (Driehuis et al., 2019). Once challenged with drugs, these tumoroids are a powerful tool to identify resistant cell populations (Campaner et al., 2020). Furthermore, several biobanks have been created (Crespo et al., 2017) and characterized by DNA and RNA sequencing (Driehuis et al., 2019), allowing drug testing before patient treatment of genetic similar tumors. Currently, the main limitation found in tumoroids is the absence of stroma and immune cells. The presence of these cells can avoid an additional step of xenograft models.

Renal tubular organoids and iPS-derived cardiac organoids showed responsiveness ti drug treatment (Guo et al., 2020; Hoang et al., 2021). Lung organoids as a model for fibrosis and SARS CoV-2 infection were used for drug testing (Kim et al., 2021; Spitalieri et al., 2022). Liver organoids are useful as disease models, but perhaps more importantly as a predictable model for drug safety. Several models have been developed (He et al., 2020), including co-culture of human hepatic progenitor and stellate cells for the simulation of a fibrotic condition (Leite et al., 2016). For drug testing these organoids must show metabolic competence, mostly evaluated by CYP induction and albumin secretion (Kim et al., 2022). Recently, a bioprinted model of human hepatocyte-like cells derived from organoids was tested under exposition of a known hepatotoxic compound, showing an expected decrease in cell viability (Bouwmeester et al., 2021).

Brain organoids are useful tools for neurodegenerative disease models. Recently, a human brain organoid model was tested for their capacity to internalize and propagate human prions. Besides, brain organoids were responsive to an established anti-prion compound, supporting their potential as a drug screening model (Groveman et al., 2021). In order to increase their reproducibility, studies have developed platforms based on automation resulting in more homogeneous organoids in terms of size, gene expression and structure compared with the pioneer protocols. However, the authors still observed a distinctly lower variability in several parameters, including survival in toxicity studies. They attributed this variability to the innate donor variability found in iPS (Renner et al., 2020). Another important limitation of organoids derived from iPS is the need for a long-term culture. These organoids must be matured for, in general, at least 30 days.

## 3D bioprinting of organoids

3D bioprinting has been used to engineer more complex and physiologically relevant tissue models. Higher resolutions can be achieved during the bioprinting process and the hierarchical organization of cells, organoids, biomaterials and growth factors that can be obtained in an automated and pre-designed way. There is also an increasing interest in the application of 3D bioprinting for the production of organoids in a high throughput system for drug screening tests (Grix et al., 2018).

Recent studies of 3D bioprinted mammary organoids in hydrogels showed a better efficiency when compared to non-bioprinted organoids (Mollica et al., 2019; Reid et al., 2019). In a model of 3D bioprinted kidney organoids, the authors showed that the bioprinting process was accurate and that the organoids had consistent nephron patterning in a large scale tissue (Lawlor et al., 2021). In a similar approach, Brassard and collaborators Brassard et al. (2021) presented a new 3D bioprinting platform named bioprinting-assisted tissue emergence (BATE). The bioprinted organoids were able to be organized as an intestinal tube tissue structure with a phenotype similar to the one found *in vivo*. Other studies have bioprinted organoids for drug screening assays (Maloney et al., 2020; Bouwmeester et al., 2021). An interesting approach was performed by Maloney and collaborators (2020); here tumor organoids were directly bioprinted in 96 well plates, allowing the drug test to be carried out without the need to transfer the organoids.

## The convergence of organoids and organ-on-a-chip: Organoids-on-a-chip

Physiological membranes are commonly recreated in microfluidic devices, using different cell types located in different sides of a porous membrane, but parenchymal tissues such as fat, liver and kidney are better replicated using complex 3D cell culture models, as for example organoids (Picollet-D'hahan et al., 2021). While organoids offer a more complex 3D model, their use is limited, due to their low throughput and reproducibility (Garreta et al., 2021).

In the pharmaceutical industry, early drug screening is based on high-throughput assay, and efficacy is first tested in 2D cell lines to discard the vast majority of compounds. Current tools for organoids development and testing are incompatible with high-throughput, making them incompatible for early drug screening, even when taking into consideration that their better prediction rates would reduce the number of tests needed to achieve reliable results. Instead, organoids assays could be used to test a relatively small number of compounds just before clinical trials. However, reproducibility must be improved for organoids, which can be achieved through biomaterials development and better control over culture environment. The pioneer protocols of organoids rely on the spontaneous stem cell differentiation and the use of Matrigel that shows bath-to-bath variability. Using bioengineering approaches, such as for example, synthetic hydrogels, desired tissue architecture can be generated from a guided stem cell differentiation, increasing reproducibility (Lancaster et al., 2017).

The culture environment of organoids at the moment suffers from the absence of vascularization, reduced organoid lifespan, and variability of tissue-specific architecture and functionality (Garreta et al., 2021). A microfluidic system includes laminar flow, therefore cells are maintained under a regular flow of cell culture medium, mimicking in part, a vascular system. Besides, the regular flow reaches a better perfusion and control of morphogen gradients compared with static cell culture, increasing cell viability and differentiation in long-term cultures. The increase of cell differentiation impacts positively on the development of tissue-specific architecture, maturation and the functionality of organoids (Homan et al., 2019; Wang et al., 2020). Besides better control of cell function, microfluidics also allows a real time monitoring of responses through sensors (Takebe et al., 2017). Microfluidic systems must ensure material consideration, including using polymers compatible with manufacturing processes, which uses thermoplastics polymers for mass-production. One recent development is the replacement of the most commonly used non-thermoplastic polydimethylsiloxane (PDMS) with an alternative biocompatible, transparent, and thermoplastic polymer, Flexdym (Lachaux et al., 2017). This allows a faster translation of scientifically validated prototype into commercially available products.

**FIGURE 1:**
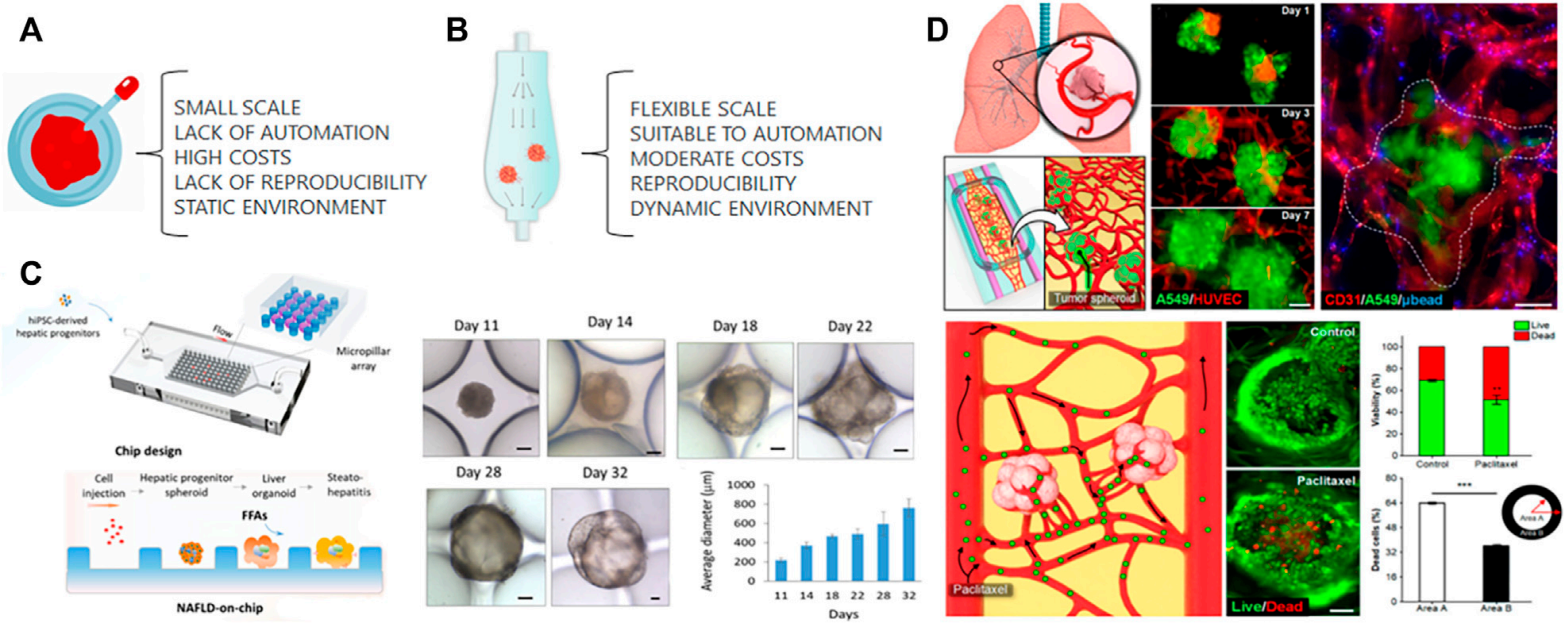
Organoids culture. **(A)** Traditional organoid culture relies on several disadvantages that can be partially solved by culturing organoids in a microfluidic chip **(B)**. The laminar flow of microfluidics provides a controlled dynamic environment increasing the reproducibility while reducing cost of organoid culture. The large scale can be reached by parallel microfluidic devices. **(C)** The cell suspension of iPS-derived hepatic progenitors cells were injected inside a microfluidic chip to form spheroids maturated into liver organoids. The maturation was followed by an increase on their average diameter during organoid culture. “Adapted with permission from Wang et al., 2020. Copyright 2020 American Chemical Society.” **(D)** Co-culture spheroids from human lung adenocarcinoma cells (A549) and endothelial cells (HUVECs) were transferred to a microfluidic device previously seeded with endothelial cells and lung fibroblasts embedded in a hydrogel. The tumor spheroids were capable of integration with the microvascular network and to drug response evaluated by Live and Dead assay. “Adapted with permission from Paek et al., 2019. Copyright 2019 American Chemical Society.” Both systems described the convergence of organoids and microfluidic, however, the increase of the complexity in **(D)** decreases its susceptibility to automation, large scale and reproducibility.

Although organoid and organ-on-a-chip pursue the same goal of mimicking tissue and organ physiology, they have emerged as 3D cell culture models disconnectedly. Organ-on-a-chip provides control and monitoring of cell functions, but are commonly simplistic models of the target organ. Organoids are based on spontaneous stem cell differentiation to recapitulate cellular and molecular events of tissue and organ formation, adding some relevant degree of variability. A synergistic strategy can address limitations and add advantages coming from both technologies. Organoids-on-a-chip will partially address the limitations of organoid models, facilitating translation to industry (Figure 1).

## Recent advances of the convergence

In recent years, advances have been made in the convergence of organoids and organ-on-a-chip technologies for drug screening, disease modeling and personalized medicine. iPS-derived liver organoids were tested on a microfluidic chip for hepatotoxicity screening. In this study, liver organoids were co-cultivated with endothelial cells and macrophages in an automated platform to seed cells, dose with drugs, collect and replenish media (Bircsak et al., 2021). The Nonalcoholic fatty liver disease (NAFLD) model was tested using iPS-derived liver organoids-on-a-chip system. These liver organoids showed upregulated expressions of lipid metabolism-associated genes in a long-term culture. These alterations indicate the abnormal lipid metabolic process found in NAFLD (Wang et al., 2020). Another organoids improvement with microfluidics includes iPS-derived kidney organoids, which are avascular and immature despite having glomerular and tubular like compartments. In millifluidic chips, kidney organoids under flow expand their pool of endothelial progenitor cells and supported angiogenesis, which in turn, improved the maturation of tubular compartments (Homan et al., 2019). Another study also showed the importance of *in vitro* vascularization. Using a perfusable 3D microvascular beds containing a co-culture of human vascular endothelial cells and fibroblasts, the authors showed that the integration of microvascular beds with other cell types recapitulates organ-specific cellular heterogeneity and structural organization of vascularized human tissues, such as adipose tissue and the blood-retinal barrier (Paek et al., 2019).

Microfluidics chips also have been tested with patient-derived organoids from solid tumors. The combination of primary human clear cell renal carcinoma with human endothelial cells in a chip results in the molecular signature of donor variation (Miller et al., 2018). In a more complex model containing patient-derived organoids and stromal cells, Haque and collaborators showed that the microfluidic chip device increased the viability of their 3D construct. Furthermore, stroma-depleting agents resulted in an increased loss of cancer cell viability in the chip device in comparison to monolayer culture (Haque et al., 2022).

Although recent advances in this field, there are still crucial limitations. Most microfluidics systems were not designed for organoids, featuring very large chambers (millifluidic chips) that accommodate together many organoids with an absence of organoid size control. When organoids are close to each other, they can fuse, forming distinct and more complex structures (Panoutsopoulos, 2021). More importantly, these chambers are not capable of forming organoids: organoids are formed first in cell culture plates and then transferred to the microfluidic device. This process, besides time consuming, increases the cost also because many organoids are lost during transfer. Current microfluidics systems designed for organoids formation are of low throughput, with only dozens of chambers per device, and too small to accommodate organoids bigger than 400 μm in diameter, making them incompatible with several organ models. In general, the process is not automated, with few exceptions (Bircsak et al., 2021).

**FIGURE 2:**
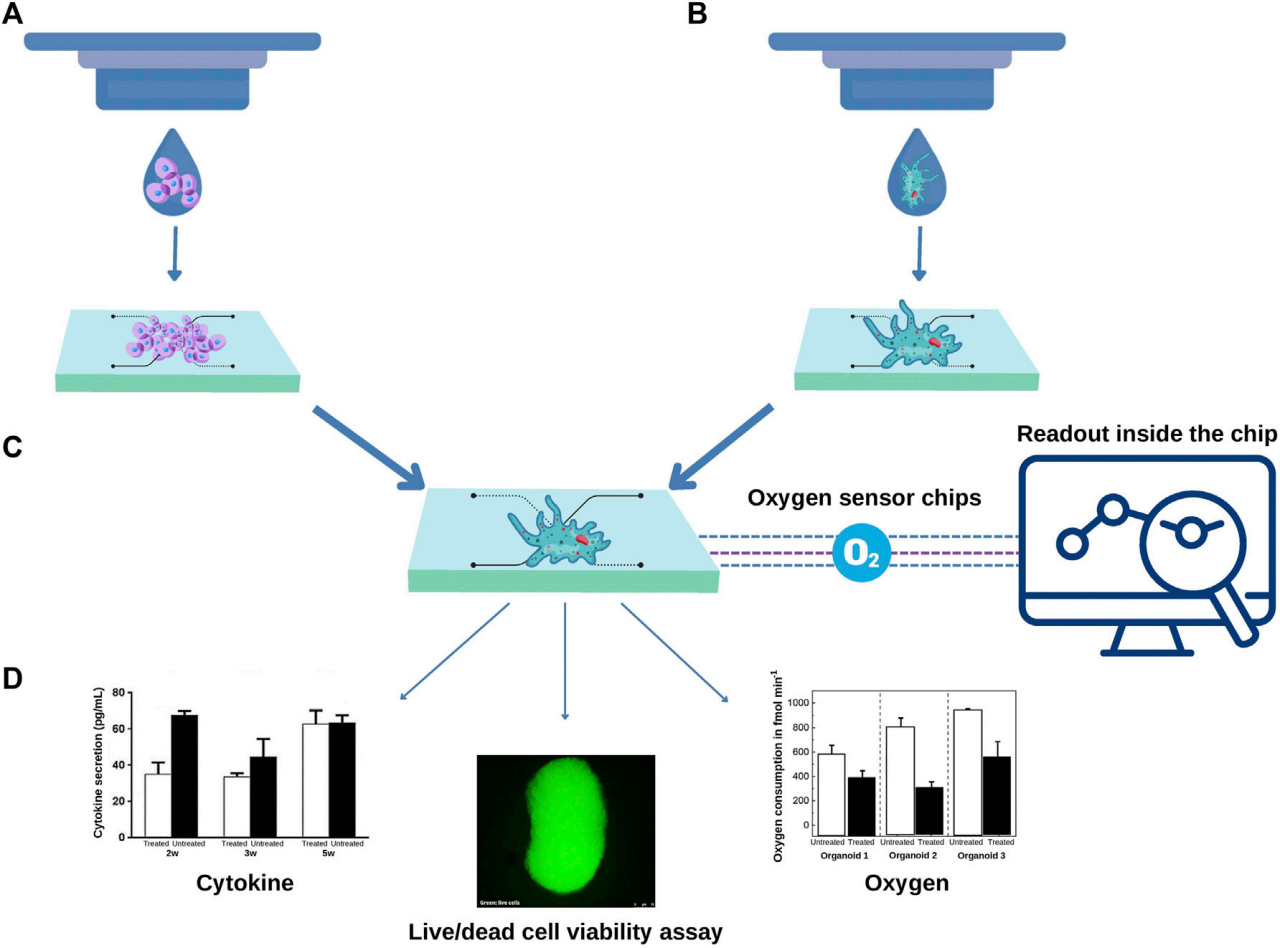
3D Bioprinting approaches can contribute to scalability of organoids-on-a-chip. **(A)** A cell suspension can be bioprinted inside a predesigned chip to form organoids or **(B)** organoids can be directly bioprinted inside a regular chip. In both scenarios the development of a specific composition of bioink according to the type of organoid is required. **(C)** The microfluidics chips can contain sensors, for example, to measure the real-time oxygen consumption from each organoid. Additional assays can be carried out inside the chip. The organoid culture supernatant can be harvested, and the levels of cytokines measured using multiplex assays. Optical transparent chips also allow fluorescent-based assays such as live and dead viability assay **(D)**.

3D bioprinting approaches could be added to different steps of the process to address the challenge of automation. For example, cell suspension can be seeded by 3D bioprinting inside the chip, providing scalability to the process. In this sense, a bioink composed of endothelial cells, smooth cells and gelatin-methacryloyl was bioprinted inside a microfluidic chip. Cells showed high viability and when compared to traditional cell culture methods, the 2D constructs had an upregulated expression of vascular proteins (Abudupataer et al., 2019). Yi and collaborators Yi et al. (2019) performed extrusion bioprinting inside an open glass chip with one inlet without perfusion. The authors used a brain decellularized bioink seeded with glioblastome or endothelial cells resulting in a circular tumor tissue with distinct layers of cells. Recently, MCF-7 spheroids were individually and precisely positioning into the microelectrode wells using a particular bioprinting approach for monitoring oxygen consumption in the absence of laminar flow (Dornhof et al., 2022). These studies open the door for more ambitious approaches, where cell suspension bioprinted inside the chips could form organoids (Figure 2).

## Advantages and limitations of the convergence

In summary, there are a set of advantages in combining organoids culture with microfluidic technologies: 1) can reduce the variability, since microfluidic devices can provide better environmental control; 2) can reduce labour cost and human error by supporting automated operation; 3) can reduce reagent use in a miniaturized culture system. This is relevant for organoids due to the high cost of recombinant growth factors used as morphogens in long-term cultures; 4) can reduce the time needed to a full maturation of organoids.

However, as an emergent field, the organoid-on-a-chip creates new technological challenges. The development of new designs for microfluidic devices is needed to generate scalable technologies and to accommodate all stages of organoid culture, since their formation until maturation. New designs imply on new methodologies for microfluidic device microfabrication. Furthermore, current microfabrication is not scalable, highlighting the needed for alternative materials.

## Concluding remarks

**FIGURE 3:**
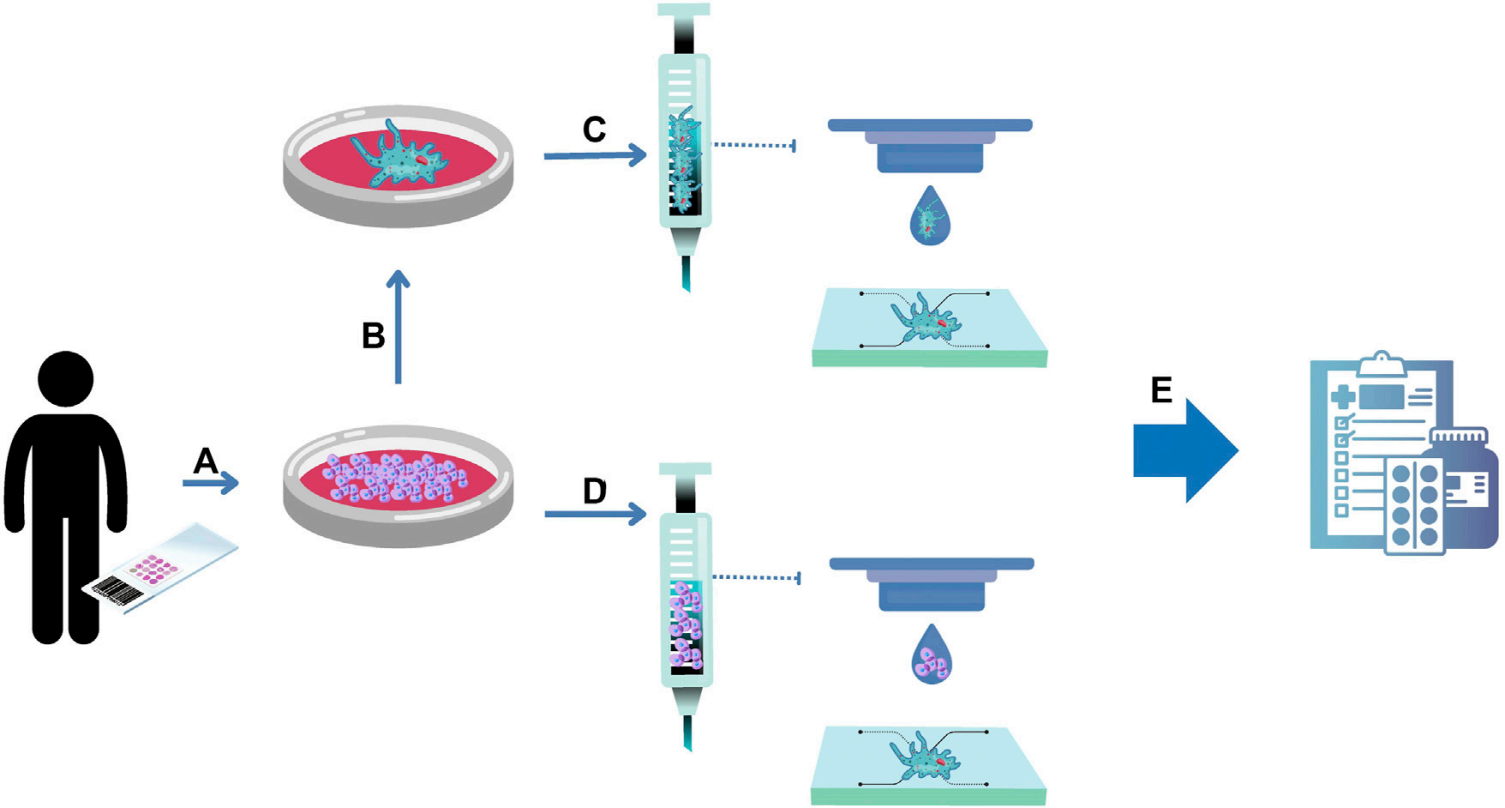
Organoids-on-a-chip for drug development. **(A)** Human primary cells can be isolated from healthy and unhealthy tissue biopsies and expanded in numbers. The resulting cells can be used to form organoids **(B)**. **(C)** Organoids can be embedded into hydrogels serving as a bioink for bioprinting protocols inside a chip. To provide scalability to the process, the resulting cells can be embedded into hydrogels serving as a bioink for bioprinting protocols to form organoids inside a chip **(D)**. The convergence of organoids and microfluidic technologies is named in this review as organoids on a chip, serving as a complex 3D organ model for drug development **(E)**.

The preclinical stage of drug development comprises 2D cell culture and animal models. Currently, there is a consensus in the scientific community that the poor predictability of such models hinders drug development and testing. Regulatory entities recognize that alternative methods, such as organoids and microfluidics, can create more reliable results, and are preparing for them to be integrated into the drug approval process. In this context, organoids emerge as a powerful technology to reduce or even replace animal models as personalized living avatars inside microfluidic chips (Figure 3).

To enable the full deployment of organoids in pharmaceutical industry, limitations around reproducibility and automation must be addressed. Microfluidic devices have already been shown to be effective for mimicking physiological barriers, drug stimulus and pathogen interactions with host cells. With organoids, microfluidics can help reduce the challenge of spontaneous differentiation of stem cells during development, providing enhanced control over spontaneous morphogenesis. Organoids-on-a-chip benefits from major advances in organoids, microfluidics, and 3D bioprinting to create models of increasing complexity, closer to their physiological counterparts. However, to reach full integration, some improvements in organoid development and microfluidics devices fabrication must be reached, with advances in 3D printing and bioprinting approaches potentially providing a high level of automation to the process.

The development of innovative, reliable, and predictable organoid-on-a-chip models of healthy and diseased tissue will have a tremendous impact on population health in the next decade. The expected breakthrough will reduce animal models and costs of drug development, adding better prediction and security to the process. We have the scientific knowledge and the technologies to reach this goal. Their integration is straightforward, but it is of the upmost importance that this technological development translates into use by the pharmaceutical industry, namely ensure high-throughput, reproducibility, and compatibility with industrial manufacturing.
