# Corrigendum: 3D organ-on-a-chip: the convergence of microphysiological systems and organoids

**DOI:** 10.3389/fcell.2024.1365671

**Published:** 2024-01-26

**Authors:** Leandra S. Baptista, Constance Porrini, Gabriela S. Kronemberger, Daniel J. Kelly, Cecile M. Perrault

**Affiliations:** ^1^ Eden Tech, Paris, France; ^2^ Universidade Federal do Rio de Janeiro, Campus UFRJ Duque de Caxias Prof Geraldo Cidade, Rio de Janeiro, Brazil; ^3^ Trinity Centre for Biomedical Engineering, Trinity Biomedical Sciences Institute, Trinity College Dublin, Dublin, Ireland; ^4^ Department of Mechanical, Manufacturing and Biomedical Engineering, School of Engineering, Trinity College Dublin, Dublin, Ireland; ^5^ Advanced Materials and Bioengineering Research Centre (AMBER), Royal College of Surgeons in Ireland and Trinity College Dublin, Dublin, Ireland; ^6^ Department of Anatomy and Regenerative Medicine, Royal College of Surgeons in Ireland, Dublin, Ireland

**Keywords:** organoids, 3D bioprinting, organ on a chip, drug development, microfluidics

In the published article, there was an error in the Funding statement. The Funding statement was not included. The correct **Funding** statement appears below.

